# Protocol to generate PDMS topographical patterns for hiPSC-derived or rat primary neuronal cultures

**DOI:** 10.1016/j.xpro.2025.104010

**Published:** 2025-08-01

**Authors:** Anna-Christina Haeb, Mireia Olives-Verger, Jordi Soriano

**Affiliations:** 1 Laboratory of Neuronal Stem Cells and Cerebral Damage, Department of Biomedical Sciences, Faculty of Medicine and Health Sciences, Institute of Neurosciences, University of Barcelona, 08036 Barcelona, Spain; 2 Department of Condensed Matter Physics, Faculty of Physics, University of Barcelona, 08028 Barcelona, Spain; 3 Universitat de Barcelona Institute of Complex Systems (UBICS), 08028 Barcelona, Spain

**Keywords:** Biophysics, neuroscience, biotechnology and bioengineering

## Abstract

Soft lithography is a promising technique to fabricate tailored substrates on polydimethylsiloxane (PDMS) casts. Here, we present a protocol to generate PDMS topographical patterns for neuronal networks *in vitro*. The protocol outlines the development of a two-level resin master mold with soft lithography and the creation of a topographical PDMS cast from the mold. We then detail the procedures for culturing either human induced pluripotent stem cell (hiPSC)-derived or rat neurons on the PDMS cast with topographical patterns and for acquiring data using calcium imaging. This protocol provides neuronal networks with imprinted connectivity.

For complete details on the use and execution of this protocol, please refer to Montalà-Flaquer et al.^1^

## Highlights

• Soft lithography to transform a black-and-white computer design into topographical molds
• A comprehensive workflow to grow neuronal cultures on PDMS topographical substrates
• Capacity to guide connectivity in neuronal cultures by changing PDMS topographical motifs
• Calcium fluorescence imaging illustrates the rich dynamics of prepared neuronal cultures

## Abstract (editor highlights)

Publisher’s note: Undertaking any experimental protocol requires adherence to local institutional guidelines for laboratory safety and ethics.

## Graphical abstract

**Figure:**
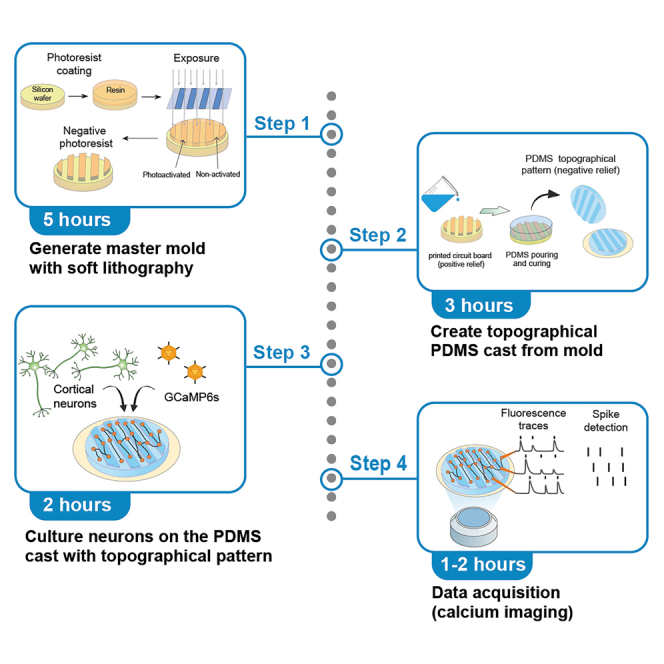

## Before you begin

Polydimethylsiloxane (PDMS) is an elastomer with excellent optical, electrical, and mechanical properties commonly used in neuroengineering.^2^ Specifically, PDMS’ transparency, thermal stability, non-toxicity, biocompatibility, and resistance to biodegradation make it ideal for generating a variety of lab-on-chip systems that include general cell culture applications, tailored microfluidic devices and engineered neuronal cultures.^1^^,^^3^^,^^4^ Here, we provide a detailed protocol for generating topographically patterned PDMS substrates to grow neuronal cultures on top of them. Our previous studies showed that topographical PDMS, as a substrate for neuronal cultures, influences network self-organization and the properties of the emerging structural connectivity, overall enriching the network’s spontaneous activity and functional characteristics, which highly contrast with those observed in cultures prepared on a flat surface.

***Note:*** In the work of Montalà-Flaquer et al.,^1^ the PDMS patterns were prepared by pouring and curing PDMS on a master mold built using printed circuit board (PCB) technology, which shapes thin copper elevations on a fiberglass substrate. To increase the spatial resolution and depth of the topographical patterns, PCB was replaced by a resin mold. The protocol described here, and its representative results, correspond to the latter design, but no differences could be observed between PCB and resin technologies in neuronal cultures grown on topographical patterns with identical characteristics.

### Institutional permissions

Dissection of rat embryonic cortices and preparation of primary neuronal cultures were carried out in accordance with the regulations of the Ethical Committee for Animal Experimentation of the University of Barcelona (approved ethical order B-RP-094/15–7125 of July 10th, 2015) and the laws for animal experimentation of the Generalitat de Catalunya (Catalonia, Spain).

### Acetate mask printing for topographical pattern fabrication on a resin mold

**Timing: 2 weeks to 1 month**

This step is necessary to transfer a computer-designed pattern onto a topographical master mold through a photolithographic process.

1. Generate the design of interest as a black and white drawing on a vector graphics editor (e.g., Inkscape or CorelDRAW), and save the design as DXF (Drawing Exchange Format) or SVG (scalable vector graphics) formats.
2. Verify the available resins for the fabrication of the master topographical mold.

***Note:*** The choice of resin (also known as photoresist), either positive or negative, will determine whether black objects in the design are transformed into elevations (positive photoresist) or depressions (negative photoresist) in the resin mold.

***Note:*** For the specific case of this protocol, the SU8 resin was used to fabricate the master mold. SU8 is a negative photoresist**,** so that black objects in the computer design become depressions in the resin mold.

***Note:*** Be aware of the difference between the resin mold and the PDMS cast. In this protocol, the cast is understood as the shape that is extracted from the mold. Thus, the PDMS cast is the topographical negative of the mold. This aspect should also be considered when designing the pattern. Thus, in the present protocol with SU8, black objects in the computer design become elevations in the PDMS cast.

3. Print the design on a suitable acetate mask with a resolution of 4,000 dpi or higher, depending on the spatial resolution of the drawn motifs and their expected height in the final master.

***Note:*** There are different options for mask materials, but the most used are acetate masks due to their ease of handling, availability and low pricing. For the results presented in this protocol, acetate masks with a resolution of 10,000 dpi were printed at JD Photo Data House (United Kingdom).

***Note:*** For lateral dimensions smaller than 50 μm or heights greater than 100 μm, it is necessary to substantially increase the mask resolution (32,000 dpi or higher), or to replace it by a chrome mask.

## Key resources table

**Table:** 

REAGENT or RESOURCE	SOURCE	IDENTIFIER
**Antibodies**		

Rabbit anti-GFAP (1:1,000)	Thermo Fisher Scientific	PA3-16727
Chicken anti-MAP2 (1:1,000)	Thermo Fisher Scientific	PA1-10005

**Bacterial and virus strains**		

GCaMP6s Ca^2+^ sensor (AAV7m8)	Viral Vector Production Unit	https://www.viralvector.eu/

**Biological samples**		

Cortical tissue from healthy E18 rat cortex tissue (CD Rat) Sprague-Dawley rat (*Rattus norvegicus*)	Charles River	N/A

**Chemicals, peptides, and recombinant proteins**		

SYLGARD 184 Silicone Elastomer Base	DWO Europe	01673921
SYLGARD 184 Silicone Elastomer Curing Agent	DWO Europe	01673921
Silicone RTV Encapsulant-QSIL 216 (PDMS alternative)	CHT	1667371
SU8 3050 resin	Kayaku Advanced Materials, Inc.	Y311075
SU8 2100 resin	Kayaku Advanced Materials, Inc.	Y111075
SU8 developer	Kayaku Advanced Materials, Inc.	Y020100
Trichloro(1H,1H,2H,2H-perfluorooctyl)silane	Sigma-Aldrich	448931
Nitric acid 65%	AppliChem	143255.1611
Laminin	Sigma-Aldrich	L2020-1MG
DPBS	Sigma-Aldrich	D8537
DAPI nucleic acid stain (1:1,000)	Thermo Fisher Scientific	D1306
Poly-D-lysine hydrobromide (PDL)	Sigma-Aldrich	A-003-M
Leibovitz’s L-15 medium	Sigma-Aldrich	L1518-500ML
MEM Eagle’s minimum essential medium	Thermo Fisher Scientific	21090022
Glutamax	Thermo Fisher Scientific	35050038
Gentamicin	Sigma-Aldrich	G1272-10ML
Glucose	Sigma-Aldrich	G5400-1KG
Horse serum	Thermo Fisher Scientific	26050088
Fetal bovine serum	Thermo Fisher Scientific	16140071
B-27 Plus	Fisher Scientific	15717988
FUDR	Sigma-Aldrich	F0503
Uridine	Thermo Fisher Scientific	A15227-14

**Experimental models: Organisms/strains**		

Sprague-Dawley rat (*Rattus norvegicus*) (embryonic brain cortices at age 18, mixed gender)	Charles River	CD rat

**Software and algorithms**		

Python version 3.12	Python Software Foundation	https://www.python.org/
Inkscape version 1.4	Inkscape Project	https://inkscape.org/
Hokawo version 2.10	Hamamatsu	https://www.hamamatsu.com/
Matlab (2018a)	MathWorks, Inc.	https://es.mathworks.com/products/matlab.html
Netcal	(Custom-made software)	https://github.com/orlandi/netcal

**Other**		

Acetate mask printing	JD Photo Data	https://www.jd-photodata.co.uk/
Petri dishes (145 mm diameter × 20 mm height)	Greiner Bio-One GmbH	639160
4-well plates (1.9 cm^2^ culture area/well)	Thermo Fisher Scientific - Nunc	179830
Branson 1800 ultrasonic cleaner	Branson Ultrasonic Corporation	CPX1800H-E
Vacuum chamber, Buerkle plastic desiccator	Thermo Fisher Scientific	10182391
Glass petri dish	Sigma-Aldrich	BR455701
Biopsy punchers	Kai Medical	BP-60F
Plasma cleaner	Harrick	PCD-002-CE
Bunsen burner (Propan)	Campingaz	CV 300 Plus
Acetate mask	JD Photo Data	N/A
Si wafer of 4″ CZ Si, N-type (phosphorous), 525 μm thick, 100 oriented, 1–10 Ωcm	Siegert Wafer GmbH	N/A
Heratherm oven	Thermo Fisher Scientific	Heratherm OMH100
Spin coater	Laurell Tech.	WS-650-23
JP Selecta Plactronic hotplate	Fisher Scientific	12022385
Mask aligner	SÜSS Microtec	MJB4
Glass cover slips Ø13 mm	Glaswarenfabrik Karl Hecht	41001113
Inverted microscope equipped for fluorescence	Zeiss	Axiovert C25
Stage top mini-incubator with CO2, multiwell plate	ibidi	Silver Line 12724
Camera Orca Flash 4.0	Hamamatsu	C11440-42U40
Syringes for filtering (50 mL)	Fisher Scientific	10636531
Filters for syringes (0.2 μm, PES membrane)	Thermo Fisher Scientific	725–2520
Nalgene rapid-flow sterile filter (0.2 μm PES, 250 mL)	Thermo Fisher Scientific	153–0020
Nalgene rapid-flow sterile filter (0.2 μm PES, 500 mL)	Thermo Fisher Scientific	566–0020

## Materials and equipment

• Antibodies:
  ○ Store them at −20°C until the indicated expiry date.
• DAPI Nucleic Acid Stain:
  ○ Store it at −20°C in the dark until the indicated expiry date.
• Mouse Laminin:
  ○ Thaw laminin at 4°C and aliquot 10 μL on ice. Store it for 3–6 months at −20°C.
• Poly-D-Lysine Hydrobromide.
  ○ Make 1 mL aliquots and store up to 12 months at −20°C.
• AVV GCaMP6s:
  ○ Perform all the steps on ice. Dilute the AVVs at 1:2 in DPBS and prepare 10 μL aliquots. Store for 3–6 months at −20°C. Prevent rethawing.
• SYLGRAD 184 Elastomer kit (base and curing agent)
  ○ Use a spoon or pipette to retrieve the base and curing agent from the original containers, weigh them according to the desired base-to-curing ratio and mix them thoroughly.
  ○ Store the Elastomer kit at room temperature until the indicated expiry date.

***Alternative:*** We recommend the QSIL 216 PDMS kit, provided by CHT Silicones (Germany).

• SU8 Resin.
  ○ Store it at room temperature until the indicated expiry date.
• Silane.
  ○ Store it in a dry and well-ventilated area in a tightly closed, chemically resistant container, away from incompatible materials.
• Nitric acid 65%
  ○ Store it in a dry and well-ventilated area in a tightly closed, chemically resistant container, away from incompatible materials, particularly solvents such as ethanol.
• L-15, MEM, Glutamax and Gentamicin.
  ○ Store them at 4°C until the indicated expiry date.
• Glucose.
  ○ Dissolve 27.023 g of glucose in 133.3 mL of Milli-Q water at 80°C with magnetic stirring at 400 rpm and then sterilize using a vacuum filter with a PES membrane (0.2 μm pore size, 250 mL). Store for 6 months at 4°C.
• Horse Serum and Fetal Bovine Serum.
  ○ Prepare 50 mL aliquots and store up to 12 months at −20°C.
• B-27 Plus.
  ○ Prepare 1 mL aliquots and store up to 12 months at −20°C.
• FUDR and Uridine.
  ○ Prepare a solution of 10 mg/mL FUDR + 25 mg/mL uridine in PBS. Make 200 μL aliquots and store up to 12 months at −20°C.

**CRITICAL:** FUDR is toxic if ingested and may irritate the eyes and skin.

**Table:** PDMS composition 10%

Reagent	Final concentration	Amount
Silicone elastomer base	90%	0.06 g/cm^2^∗
Silicone elastomer curing agent	10%	0.006 g/cm^2^∗

∗Quantity for a Petri dish.

**Table:** Coating

Reagent	Final concentration
Laminin	5 μg/mL
Poly-D-lysine	100 μg/mL

**Table:** Resin mold generation

Process	SU8 3050	SU8 2100
Spin Coating	1) 10 s at 500 rpm and 100 rpm/s 2) 30 s at 3300 rpm and 300 rpm/s	1) 10 s at 500 rpm and 100 rpm/s 2) 30 s at 3100 rpm and 300 rpm/s
Soft Bake	20 min at 95°C	1) 5 min at 65°C 2) 20 min at 95°C
Exposure process	250 mJ/cm^2^	240 mJ/cm^2^
Post Bake	1) 1 min at 65°C 2) 5 min at 95°C	1) 5 min at 65°C 2) 10 min at 95°C
Hard bake	1) 30 min at 95°C 2) 10 min at 65°C

**Table:** L-15 medium

Reagent	Final concentration	Amount
L-15 media	1×	500 mL
Glucose	1 M	16.6 mL
Gentamicin	1×	1 mL
Total	N/A	517.6 mL

***Note:*** Inside a chemical hood, bubble L-15 in a sterile bottle for 20 min in O_2_ (use a clean new pipette and an O_2_ tanker, protecting the opening of the bottle with parafilm). After bubbling, sterilize the medium using a vacuum filter with a PES membrane (0.2 μm pore size, 500 mL). Repeat the bubbling and filtration process every 15 days. Store the medium at 4°C for up to 3 months.

**Table:** MEM + 3G

Reagent	Final concentration	Amount
MEM	1×	500 mL
Glucose	1 M	10 mL
Glutamax	1×	5 mL
Gentamicin	1×	2 mL
Total	N/A	517 mL

***Note:*** Sterilize using a vacuum filter with a PES membrane (0.2 μm pore size, 500 mL). Store at 4°C for up to 3 months.

**Table:** Plating medium

Reagent	Final concentration	Amount
MEM + 3G	N/A	45 mL
Horse Serum	1×	2.5 mL
Fetal Bovine Serum	1×	2.5 mL
B-27 Plus	1×	50 μL
Total	N/A	50.05 mL

***Note:*** Sterilize using a 0.2 μm pore size syringe filter. Store at 4°C for up to 2 weeks.

**Table:** Changing medium

Reagent	Final concentration	Amount
MEM + 3G	N/A	45 mL
Horse Serum	1×	5 mL
FUDR+URIDINE solution	N/A	200 μL
Total	N/A	50.2 mL

***Note:*** Sterilize using a 0.2 μm pore size syringe filter. Store at 4°C for up to 2 weeks.

***Note:*** The purpose of adding the FUDR+URIDINE solution is to reduce the proliferation of glia, a family of supporting cells that are very important during early development of the neuronal culture.

**Table:** Final medium

Reagent	Final concentration	Amount
MEM + 3G	N/A	45 mL
Horse Serum	1×	5 mL
Total	N/A	50 mL

***Note:*** Sterilize using a 0.2 μm pore size syringe filter. Store at 4°C for up to 2 weeks.

## Step-by-step method details

Here we provide the protocol to generate the PDMS patterns, as well as the protocol to sterilize glass coverslips. It is recommended to start the coverslip cleaning during the 60 min curation of the PDMS in the oven (PDMS preparation, step 18).

### Resin mold generation: Photolithography process

**Timing: 5 h**

Here, we describe the steps to generate a high-quality resin master mold with the desired pattern and height.

***Note:*** The master mold only needs to be fabricated once and can then be reused for future experiments. The hardness and stability of both the silicon wafer and SU8 resin allow the mold to withstand hundreds of PDMS casting cycles. The key factors for the longevity of the mold are the meticulous peeling of PDMS and the periodic resin re-silanization to ensure clean separation between the resin and PDMS.

***Note:*** The following steps must be carried out in a clean room.

1. Use a silicon wafer as the base material for the fabrication of the pattern.
2. Desiccate the wafer at 200°C for 10 min using a hot plate.
3. Perform an oxygen plasma treatment on the wafer for 30 s at high power and 0.8 Torr pressure.

***Note:*** This step eliminates organic materials and makes the surface hydrophilic for better resin adhesion.

4. Spin coating: deposit a uniform thin film of resin on the silicon wafer.

***Note:*** The choice of resin type depends on the desired height of the patterns (Limitation 1). For the specific case of this protocol, SU8 3050 was used to achieve a height of 50 μm and SU8 2100 to achieve a height of 100 μm.

***Note:*** The corresponding spin coating parameters are: For SU8 3050 resin, first 10 s at 500 rpm and 100 rpm/s acceleration, then 30 s at 3,300 rpm and 300 rpm/s; for SU8 2100 resin, first 10 s at 500 rpm and 100 rpm/s, then 30 s at 3,100 rpm and 300 rpm/s.

**CRITICAL:** The deposition of a uniform thin film of resin and the specific values of rpm and acceleration are important for achieving consistent uniform pattern height and quality across the wafer.

5. Soft bake: leave the wafer on the hot plate for a specific period of time, depending on resin.

***Note:*** This step removes solvents and hardens the resin.

***Note:*** For the specific case of this protocol, the parameters are: For SU8 3050 resin, 20 min at 95°C; for SU8 2100 resin, first 5 min at 65°C and then 20 min at 95°C.

6. Exposure process:
  a. Use a mask aligner to transfer the patterns to the wafer through a shadow transfer method.
  b. Place the photomask (acetate film with the patterns) between a UV light source and the target wafer.

***Note:*** In this way, the resin deposited on the wafer, as a photosensitive material, reacts to the light projected from the image, creating both photoactivated and non-activated regions. The non-activated regions are the black elements of the acetate mask.

***Note:*** For the specific case of this protocol, the illumination energy parameters are: 250 mJ/cm^2^ for SU8 3050 resin; and 240 mJ/cm^2^ for SU8 2100 resin.

**CRITICAL:** The illumination energy and the alignment of the photomask are essential for accurate pattern definition. Insufficient or excessive exposure, or misalignment, will result in pattern distortions or feature loss.

7. Post bake: leave the wafer on the hot plate for a specific period on time.

***Note:*** This step completes the chemical reaction initiated by UV exposure, which activates the photoactive compound in the photoresist, and to ensure a uniform distribution of the activated sites.

***Note:*** For the specific case of this protocol, the parameters are: For SU8 3050 resin, first 1 min at 65°C and then 5 min at 95°C; for SU8 2100 resin, first 5 min at 65°C and then 10 min at 95°C.

***Note:*** Remember that the spin coating parameters as well as the soft bake, exposure, and post bake times depend on the selected resin.

8. Development of photoresist: submerge the wafer in a developer solution.

***Note:*** This step removes either the photo-activated resin (for positive photoresist) or the non-activated resin (for negative photoresist).

**Figure 1:**
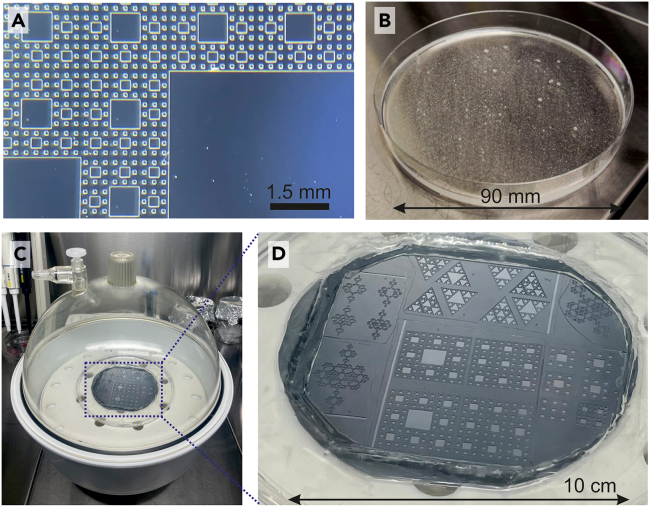
From resin mold to final topographical patterns on PDMS (A) Detail of a topographical pattern on a resin mold. Each outlined square is a depression in the pattern. (B) Liquid PDMS on a Petri dish after mixing the base with the curing agent. (C) Resin mold covered with liquid PDMS, placed inside a vacuum chamber to eliminate air bubbles. (D) Detail of the PDMS-covered mold, absent of bubbles.

***Note:*** In this way, specific regions of the silicon wafer surface are cleaned off and exposed, shaping a topographical wafer-resin master mold that replicates the pattern of the photomask (Figure 1A).

***Note:*** The development time depends on the resin type. The larger the desired height of the patterns, the longer it must be kept in the solvent.

**CRITICAL:** The development time is important; insufficient development will result in residual unwanted resin, while over-development can lead to the erosion of delicate features.

9. Hard bake: Leave the resin on the hot plate for 30 min at 95°C and 10 min at 65°C.

***Note:*** This process improves thermal and mechanical properties and removes any residual solvents, ensuring the integrity of the pattern during subsequent processing steps.

10. Wafer silanization: Apply a hydrophobic silane coating by vaporizing a few microliters of silane in a vacuum chamber that contains the resin surface.

***Note:*** The hydrophobic nature of silane substantially improves the demolding of the PDMS cast and therefore ensures a high topographical fidelity from the mold to the cast.

**CRITICAL:** Silane is hazardous and requires strict safety precautions. Always work under a chemical fume hood and consult the Safety Data Sheet (SDS) to ensure proper manipulation.

**CRITICAL:** Effective silanization is critical for the longevity and reusability of the resin mold. Silanization prevents PDMS adhesion, thereby protecting delicate features and extending the mold's lifespan.

### PDMS preparation

**Timing: 3–4 h**

Here, we describe the steps to generate the PDMS cast from the resin master mold.

***Note:*** Since the PDMS reagents are not sterile and environmental particles such as dust can easily stick to PDMS, the below procedures should be carried out in a laminar flow hood or in a clean room to provide additional sterile conditions. In the Glass coverslip cleaning, the PDMS will be autoclaved to ensure sterility (problem 1).

11. Place the resin mold on a sterile, plastic circular petri dish.

***Note:*** For this protocol, the resin mold was 80 × 80 mm^2^ in size and was placed on a 145 mm diameter Petri dish.

12. PDMS is prepared by mixing an elastomer base and a curing agent with typically a 10:1 ratio (elastomer to curing agent), with a total mass sufficiently high to cover the resin mold.
  a. To prepare the mixture, pour the elastomer base using a disposable plastic pipette and weigh it inside a sterile disposable container, e.g., a small petri dish (90 mm diameter × 20 mm height).
  b. Then, add the curing agent, and thoroughly mix the materials.

***Note:*** The PDMS elastomer is highly viscous and therefore it must be pipetted up very slowly. If a pipettor is used instead of a plastic pipette, be sure to use filter tips to prevent damage in case the elastomer is accidentally drawn into the pipettor.

***Note:*** In this protocol, we considered 60 mg/cm^2^ of elastomer and 6 mg/cm^2^ of curing agent. This 10:1 ratio can be modified to consider softer PDMS substrates, e.g., by using 30:1 or 100:1 ratios. Different studies have shown that PDMS softness influences neuronal development and network connectivity^5^^,^^6^^,^^7^ (Limitation 2).

13. Thoroughly mix the liquid PDMS and ignore the formation of air bubbles. Stop mixing when the PDMS achieves a whitish color and that indicates a high presence of bubbles (Figure 1B).

**CRITICAL:** Thorough mixing of the PDMS elastomer and curing agent is essential to ensure a uniform cross-linking and complete curing of the PDMS cast. Inadequate mixing may result in regions with incomplete curing, causing variations in mechanical properties and potential structural weaknesses.

14. Place the container with the liquid PDMS on a pre-cleaned vacuum chamber and apply a vacuum pressure of 60 Torr for 15 min (Figures 1C and 1D).

**CRITICAL:** Effective bubbles removal is crucial for producing a structurally uniform PDMS cast, ensuring high fidelity transfer of the resin’s topographical features, and preventing surface irregularities that could compromise cell culture procedures.

15. Slowly release the vacuum inside the chamber and inspect the mixture for air bubbles.
  a. In case that bubbles remain, typically on the surface of the PDMS, use a new Pasteur pipette to create an air stream that is directed onto the air bubbles.
  b. If too many bubbles are still present, repeat step 14 with higher vacuum pressure and time.
16. Slowly pour the PDMS mixture over the resin mold. Perform this step on a flat surface to ensure an even spread of the PDMS and a uniform coverage of the mold.

***Note:*** We recommend preparing a ‘control cast’ (without topography) by pouring and curing PDMS directly on a sterile plastic Petri dish to create a flat surface.

***Note:*** Be aware that the poured mass of PDMS will define the main thickness of the cured PDMS cast. The poured mass must be therefore adjusted to fit the desired experimental application. For imaging neurons (see recording neuronal activity), the PDMS cast should be as thin as possible without making it too fragile. An adequate thickness is around 0.5 mm.

17. Repeat steps 14–15 to remove the air bubbles generated during the PDMS pouring process.
18. Place the petri dish containing the resin and PDMS in an oven at 70°C for 1 h.

***Note:*** The time and temperature of curing are crucial for the final PDMS mechanical properties. The longer the time and the higher the temperature, the stiffer the PDMS cast will be. Neuronal attachment and development substantially depend on whether the PDMS is soft or stiff,^5^^,^^6^^,^^7^ with stiffer substrates leading to shorter neurites. Thus, the structure of the formed neuronal network or its dynamics may change depending on the mechanical properties of the PDMS (Limitation 2).

**Figure 2:**
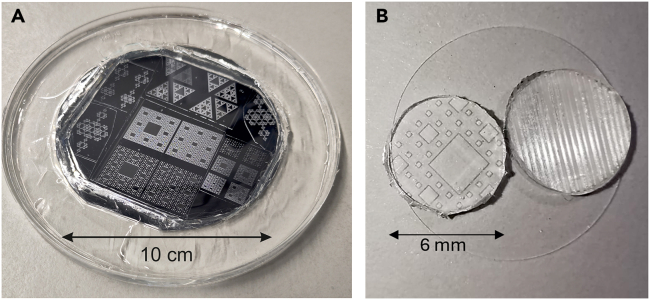
PDMS cast for cell culturing (A) Cured PDMS on top of the resin mold ready for unmolding. The depressions (elevations) of the mold become crevices (valleys) in the PDMS cast. (B) A pair of PDMS casts containing the desired topographical pattern on top of a glass coverslip, ready for cell culturing.

19. Let the cured PDMS (Figure 2A) cool down and gently unmold the PDMS from the resin, finally obtaining a PDMS cast with topographical motifs.
20. Use a clean scalpel or a puncher with the desired diameter (for example, 6 mm) to cut out a PDMS disc. The PDMS pattern is ready to be attached to a glass coverslip (Figure 2B) (problem 1).

### Glass coverslip cleaning

**Timing: 3.5 h**

In the following steps we describe the cleaning of the coverslips from dust and residues to keep the cell culture free from contamination.

21. Place the glass coverslips via a holder in 65% nitric acid for 90 min at room temperature.

**CRITICAL:** Due to the toxicity of nitric acid, perform this step in a fume hood. Consult SDS for proper manipulation.

22. Take out the coverslips from the nitric acid and wash them twice with double distilled (Milli-Q) water.

***Note:*** This is carried out by placing the holder with the coverslips into two consecutive containers filled with Milli-Q water.

**CRITICAL:** This step prevents an exothermic reaction of nitric acid with ethanol, used next, that would produce toxic fumes and potentially explosive compounds.

23. Place the holder with the coverslips in 70% ethanol and sonicate them for 20 min.

**CRITICAL:** The cleaning steps involving nitric acid, Milli-Q water and ethanol immersion are important for the effective removal of both inorganic and organic contaminants from the glass coverslip surfaces. To maintain optimal cleaning efficacy, the periodic replacement of the nitric acid and ethanol solutions, approximately every two weeks, is strongly recommended due to the potential contaminant accumulation that may compromise their cleaning capabilities. In addition, it is of utmost importance to ensure the complete elimination of residual nitric acid prior to exposure to ethanol, because the combination of these substances can result in a hazardous chemical reaction.

24. Carefully dry the coverslips using a Bunsen burner and place them in a sterile glass petri dish.
25. Place the previously punched PDMS casts onto the coverslips.

***Note:*** For the representative results presented in this protocol, a pair of 6 mm PDMS patterns is placed on a 13 mm glass coverslip (Figure 2B) (problem 2).

26. Autoclave the PDMS-coverslip assembly to ensure sterilization and facilitate a firm attachment of the PDMS to the glass surface.

***Note:*** The autoclaving parameters used in this protocol are: 105°C temperature, 25 min sterilization time, 15 min drying time, and a single cycle (problem 1).

**CRITICAL:** The autoclaving of the PDMS-coverslip assembly constitutes a critical sterilization procedure necessary for the elimination of microbial contamination and for facilitating a robust bond between the PDMS and the glass coverslip.

27. Place the PDMS-coverslip assemblies on a 4-well plate in preparation for culturing.

***Note:*** The patterned substrates are now ready for coating and to host a neuronal network.

### Culturing cells atop the PDMS-patterned substrates

**Timing: 2 days**

The following steps describe the preparation of rat primary neuronal cultures on the surface of the topographical patterns and the quantification of neuronal activity via fluorescence calcium imaging, which enables the validation of healthily neuronal growth on PDMS and the confirmation that neurons’ collective behavior is influenced by the imprinted topography.

**CRITICAL:** Careful plan your cell culture model (e.g., rat primary or human-derived neuronal cultures^8^) to properly adapt the following steps, particularly regarding adhesive protein coating and cell seeding density. When working with human induced pluripotent stem cells (hiPSCs)-derived neurons, the preparation of the cell culture will take longer, around 3–4 weeks, depending on the protocol,^8^^,^^9^ due to thawing, maintenance, and differentiation of the cells.

***Note:*** In the present protocol we consider rat primary cortical cultures derived from rat embryos at embryonic day 18 (E18). This developmental stage is considered ideal since all cortical layers are formed but are not fully connected yet, allowing mechanical dissociation to effectively produce a single-cell suspension. Gliogenesis begins at E16, leading to the emergence of astrocytes.^10^^,^^11^^,^^12^ Consequently, the E18 cortical cell suspension contains both cortical neurons (from all layers) and a small number of astrocytes. In contrast, cell extractions performed before E18 would contain fewer neurons and astrocytes, while tissues from older embryos or postnatal brains would require a combination of chemical and mechanical dissociation due to established brain connectivity, substantially increasing cell death and tissue debris.

28. Carry out a plasma treatment for 30 s at 0.8 Torr.

***Note:*** This step modifies the wettability of the PDMS from hydrophobic to hydrophilic (problem 3).

**CRITICAL:** For PDMS topographical patterns featuring microscale motifs, the plasma treatment of the PDMS surface is a crucial step to ensure a uniform coating of adhesive proteins.

29. Coat the PDMS topographical substrates with adhesive proteins.
  a. Add 1 mL of the adhesive protein PDL into the wells containing the PDMS patterns.
  b. Incubate overnight at 37°C.

***Note:*** This step allows the uniform growth of neuronal cells on top of the PDMS surfaces (problem 4).

***Note:*** This coating should be carried out within 60 min of plasma treatment to ensure that surface hydrophilicity is preserved.

***Note:*** The coating may differ depending on the used cell model, e.g., rat primary neuronal cultures or hiPSCs-derived neuronal cultures, as illustrated in Estévez-Priego et al.^8^

**CRITICAL:** The coating of the PDMS substrates with adhesive proteins is essential to promote neuronal adhesion. Neurons exhibit limited affinity to untreated PDMS surfaces and tend to form aggregates in the absence of such coatings (problem 3).

30. Next day, remove residual proteins in the wells as follows:
  a. Wash the wells twice with sterile Milli-Q water.
  b. Then, wash once with plating medium.
  c. Finally, fill the wells with 1 mL of fresh plating medium warmed at 37°C.
31. Add 1 mL of suspended cells in plating medium into the wells, covering the PDMS substrates homogeneously.

***Note:*** The typical nominal density of plated cells is about 1,600 cells/mm^2^.

***Note:*** The day of culturing the cells represents day *in vitro* (DIV) 0.

***Note:*** The full culturing procedure takes 2 h and includes cortex dissection in ice-cold L-15 medium followed by mechanical dissociation in plating medium through repeated pipetting until a single cell suspension is achieved.

32. Infect the cultures with adeno-associated virus (AAVs) carrying the GCaMP6s genetically encoded Ca^2+^ indicator under the control of the synapsin-I promoter.

***Note:*** Synapsin-I is a neuron-specific phosphoprotein whose expression levels correlate with neuronal maturation.^13^^,^^14^ Consequently, only mature neurons are imaged.

***Note:*** We use the AAV7m8 serotype to deliver the GCaMP6s construct into the cells given its enhanced GFP expression in *in vitro* cortical networks, including both rat primary and hiPSC-derived neuronal cultures.^15^

**CRITICAL:** Follow biosafety procedures when working with AAVs (biosafety level 1). Infect the cells in a properly certified biosafety cabin and wear appropriate personal protective equipment. Further, be aware that 70% ethanol cleaning will not inactivate viruses. Thus, surfaces should be disinfected with 10% bleach to ensure proper decontamination.

33. At DIV 4, replace the plating medium by changing medium to limit the growth of glial cells.
34. At DIV 7, replace the changing medium by final medium.
35. Refresh the final medium every 3 days until the end of measurements by replacing the entire culture well volume.

### Recording neuronal activity

**Timing: 1 h**

Spontaneous neuronal activity can be monitored through fluorescence calcium imaging from DIV 5–6 (onset of fluorescence) to typically DIV 21 (Limitation 3). For the acquisition of neuronal activity data, we use an inverted microscope equipped with a high-speed CMOS camera and a fluorescence imaging system^16^ (problem 5). Activity is recorded in our representative experiments using Hokawo software, a Hamamatsu-dedicated imaging package designed for camera control and image acquisition.

***Note:*** After seeding and infecting the neurons, it takes about 5–6 days for neurons to mature and express the GCaMP6s fluorescent Ca^2+^ indicator with sufficiently strong fluorescence, which is fundamental to monitor their activity with high signal-to-noise ratio. Thus, DIV 6 represents the earliest time point for experimental measurements in rat primary cultures, and experimental timelines should be planned to accommodate this 5-day window.

***Note:*** hiPSC-derived neuronal cultures require 14–21 DIV post-differentiation to achieve indicator expression levels comparable to those of rat primary cultures. Additionally, neuronal maturation and thus the expression of the calcium indicator vary between rat and hiPSC-derived neurons. A comprehensive comparison of the differences in development and spontaneous activity of rat primary and hiPSC-derived neuronal cultures, specifically in the context of fluorescence calcium imaging, is provided in Estévez-Priego et al.^8^

***Note:*** As alternative to genetically encoded Ca^2+^ indicators (GECIs), a 20 min incubation with a chemical Ca^2+^ indicator for live-cell staining, such as Fluo-4-AM, can be used to record neuronal activity.^16^ However, the advantage of GECIs for neuronal activity is that neuronal behavior can be monitored throughout culture’s whole development, whereas live-stained cultures can be used only once.

36. Take the neuronal culture from the incubator and place it under the microscope.

***Note:*** For an optimal outcome, recordings should be performed inside a transparent mini-incubator at 37°C, 5% CO_2_ and 95% humidity to preserve physiological conditions. Let the cells adapt for 10 min.

37. Record neuronal activity for 10–30 min, depending on the experimental goal.

***Note:*** A 10 min recording is sufficient to assess the overall behavior of PDMS-grown neuronal cultures along development.^8^ or after chemical perturbations, whereas 30 min recordings may be needed for those quantifications in which abundant activity statistics is important, for instance for functional connectivity analyses or the characterization of spatiotemporal fronts of neuronal activity.^1^

**CRITICAL:** Recordings should be performed in complete darkness since external light (e.g., laboratory lighting) may add artifacts to the recorded signal (problem 5).

38. Analyze neuronal activity from recorded image sequences.
  a. Identify regions of interest (ROIs) in the recorded image sequences using a commercial software or custom-made analysis tools (e.g., the software Netcal^17^).
  b. Then, extract the average fluorescence within each ROI a long time.
  c. Finally, infer neuronal activity events (spike trains) from the fluorescence signal, typically by ascribing sharp increases of the fluorescence trace to an action potential or a burst of them.

***Note:*** Inferring spikes from fluorescence traces is challenging and can be carried out using different approaches, from simple thresholding of the fluorescence signal to detailed biophysical models of signal dynamics. The resulting data and subsequent analysis may therefore vary depending on the approach employed.^16^^,^^17^

## Expected outcomes

**Figure 3:**
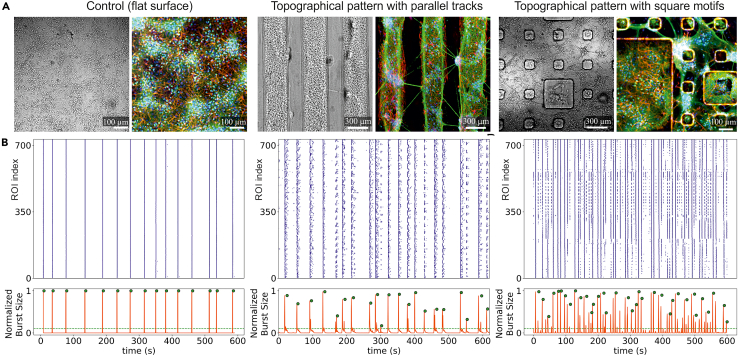
Homogeneous and topographically modulated rat primary neuronal cultures (A) Bright-field and immunostaining images of a homogeneous neuronal culture on a control flat PDMS surface (left), one grown on a topographical PDMS surface shaped as parallel, periodic elevations termed *tracks* (center), and another one shaped as randomly positioned square elevations (right). Images correspond to day *in vitro* (DIV) 7. Cultures are prepared in all cases with a homogeneous distribution of neurons on PDL-coated substrates, but aggregation in some areas may appear as the cultures develop. For the immunocytological staining, green shows MAP2-positive neurons; red shows GFAP-positive glial cells; and blue the cell nuclei. (B) Analysis of spontaneous activity recordings obtained through fluorescence calcium imaging for the three culture preparations, showing the raster plot of activity (top) together with the size of bursting events (bottom). The raster plot indicates the time of activation of each region of interest (ROI) in the culture, in this case neurons, whereas the size of bursting accounts for the tendency of neurons to activate together in a short time window, with a bursting size of 1 corresponding to the coherent activation of all neurons in the culture.

To demonstrate the effect of the topographically modulated PDMS surfaces on rat neuronal network morphology, Figure 3A compares bright-field and immunocytological images at DIV 7 of a homogeneous (left) and two topographically-modulated PDMS surfaces characterized by *tracks* (center) and *squares* motifs (right). In all cases, PDMS surfaces were treated with oxygen plasma and coated with PDL. The *tracks* and *squares* configurations correspond to the designs that were explored in detail in Montalà-Flaquer et al.,^1^ showing that the connectivity and dynamics of the prepared cultures depended on the underlying topography. In general, for the PDMS topographical cultures, different populations of cells grow on either the top or bottom of the motifs, with neurites often following the contour of the PDMS patterns. We note that some cultures exhibit neuronal aggregation, a characteristic that frequently appears on the topographical surfaces as compared to flat ones, and that is caused by the pulling forces of the neurites during network formation in combination with the spatial anisotropy imposed by the PDMS. Aggregation can be reduced by combining different adhesive proteins that strengthen cell attachment to the surface (problem 3).

The recording of neuronal spontaneous activity through Ca^2+^ imaging allows us to compare the dynamics between homogeneous and topographical neuronal networks. Figure 3B shows raster plots of activity for the three representative rat primary neuronal cultures together with the population activity, which quantifies the size of network bursts, i.e., the coherent activation of a large fraction of the neuronal network in a short time window. While the homogeneous network activates in strong quasi-periodic bursts that encompass the entire network, the PDMS topographical ones exhibit a much richer repertoire of bursting sizes and timings. Details of the statistical quantification of neuronal activity for these PDMS cultures are provided in Montalà-Flaquer et al.^1^

**Figure 4:**
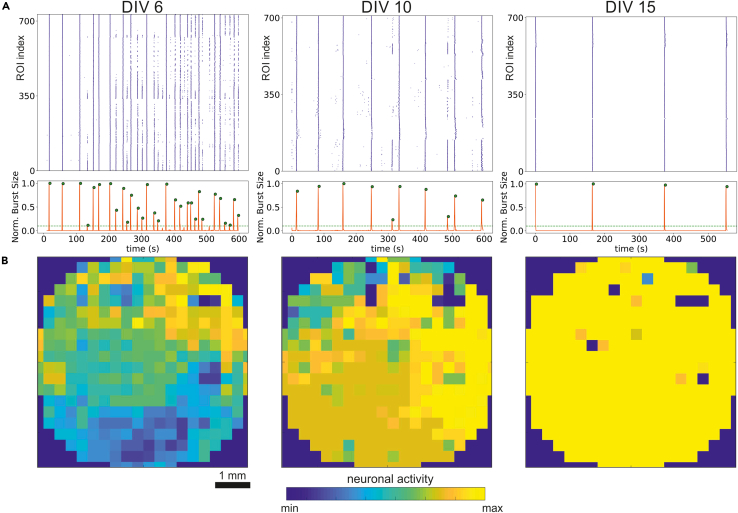
Changes in the dynamics of PDMS-grown neurons during development (A) Sequence of raster plots (top) and bursting sizes (bottom) for a rat primary neuronal culture prepared on a PDMS topographical substrate shaped as square motifs. The same neurons are monitored along different days, from the onset of GCaMP6s expression to maturation. The network dynamics changes during development, evolving from small-sized collective activations at DIV 6 with no network-wide bursting, to a balanced regime at DIV 10 in which network-wide bursts may appear but are rare, and finally to a bursting-dominated regime at DIV 15 in which all neurons activate at unison. (B) Corresponding images of the distribution of network activity across the network, illustrating that activity is heterogeneous and modulated by the PDMS motifs at young network stages, but not in mature ones by DIV 15, in which all neurons always activate together.

GCaMP6s expression in the rat primary neuronal cultures enables the monitoring of neuronal activity in the same neurons over long periods of time (Figure 4). Early neuronal activity corresponds to DIV 6, a regime characterized by highly localized activations strongly influenced by the PDMS motifs. Following, DIVs 7–14 show a balanced regime, in which both local and global activations coexist. At later time points, neurites may easily extend beyond the characteristic PDMS motifs’ size, erasing their capacity to constrain neuronal connectivity and shaping a highly coherent network dynamics, i.e., dominated by network-wide bursting. Thus, one could consider DIVs in the range 7–14 as optimal when investigating the emergence of rich network activity or structure-to-dynamics relationships. This continuous evolution of the neuronal cultures was treated in detail in the original work of Montalà-Flaquer et al.^1^

## Limitations

### Limitation 1: Wafer height

The quality of the mask used in the wafer fabrication process significantly affects the achievable wafer height and lateral pattern size. Acetate masks, characterized by medium-low spatial resolutions in the range 4,000–10,000 dpi, generally limit lateral pattern sizes to approximately 50 μm and restrict maximum wafer heights to around 100 μm. In addition, at this maximum height, resin removal from smaller regions can be difficult, which can lead to imperfect wafers (resin mold generation: Photolithography process).

### Potential solution

A first possibility is to design patterns with larger lateral sizes when possible. If lateral detail is critical, a second possibility is to improve the resolution of the acetate mask. For example, designs with lateral spatial dimensions of 20–30 μm and heights of around 150–200 μm require an acetate mask resolution of at least 40,000 dpi. For wafers requiring heights greater than those achievable with acetate masks, it is recommended to use a chrome mask.

### Limitation 2: PDMS base-to-curing agent ratio

The prepared PDMS cast must be unmolded from the resin mold before use. Excessively stiff PDMS may easily break into big fragments during the unmolding process, whereas excessively soft PDMS may be sticky and leave behind tiny fragments on the resin’s deepest areas upon unmolding. Since the stiffness of the PDMS critically depends on the base-to-curing agent ratio, the mechanical behavior of the produced PDMS cast may vary when this ratio is changed, e.g., from the standard 10:1 to a highly soft 100:1 or a highly stiff 3:1 ratio. Additionally, PDMS stiffness not only affects the easiness for unmolding but also alters the development of the neuronal network growing on it. Different studies have reported that the capacity of axons to extend across a substrate and connect to other neurons depends on the stiffness of the substrate itself.^5^^,^^6^^,^^7^ Thus, the resulting networks and their dynamics may differ from the representative results presented here (PDMS preparation).

### Potential solution

Carefully plan ahead which is the desired stiffness of the PDMS cast. Preliminary tests on just flat PDMS may help tune the optimal mechanical characteristics for specific experiments. When considering PDMS topographical substrates, it may be useful to first fabricate molds with relatively large lateral objects to evaluate the easiness of manipulation and unmolding. In this regard, silane coating is crucial for adequate unmolding and should be carried out. On the other hand, when growing neurons and analyzing the obtained data, be aware that the observed results may depend on the considered PDMS stiffness, particularly when comparing the results with other works in the literature.^5^^,^^6^^,^^7^ In our case, we observed that a base-to-curing agent ratio increase from 10:1 to 3:1 raised the Young’s modulus of the PDMS from 2 MPa to 6 MPa, i.e., made the PDMS stiffer. Neurons growing on this stiffer substrate exhibited shorter axons and displayed a dynamic behavior that was characterized by a reduction in the frequency of neuronal bursting events.

### Limitation 3: Culture maturation promotes network interconnection

Axonal length and overall network connectivity increase as the neuronal cultures mature on the PDMS topographical patterns. The increased interconnectivity gradually smooths out the local anisotropies imprinted by the topographical patterns, overall shifting neuronal network dynamics from localized activity to persistent network-wide bursting typical of homogeneous substrates, as illustrated in Figure 4.

### Potential solution

There is no optimal solution to this problem, and it is indeed the focus of extensive research given the interest to design neuronal cultures with precisely imprinted connectivity features that remain stable. However, a possible mitigation would be to increase the depth of the topographical pattern so that local anisotropies are retained for longer time periods. However, the experimentalist should be aware that it is not possible to control neuronal culture development or to freeze the connectivity at a desired time point. Thus, intensive testing should be carried out to ascertain the best time point for the desired dynamic behavior. In our explorations (Figure 4), DIV 5–6 represents the earliest time point for experimental measurements, which coincides with the expression of GCamP6s and the overall presence of short neurites, shaping a network dynamics characterized by highly localized activations strongly influenced by the PDMS motifs. DIVs in the range 7–14 correspond to a balanced regime, in which both local and global activations coexist. At later time points, neurites may easily extend beyond the characteristic PDMS motifs’ size, erasing their capacity to constrain neuronal connectivity and shaping a highly coherent network dynamics, i.e., dominated by network-wide bursting. Thus, as Figure 4 illustrates, one could consider DIVs in the range 7–14 as optimal when investigating the emergence of rich network activity or structure-to-dynamics relationships.

## Troubleshooting

### Problem 1: Sterility and biocompatibility of PDMS

Non-sterile PDMS represents a high risk for contamination. Especially in long-term cell-culture and in cells treated without antibiotics, such as hiPSC-derived neuronal cultures, which have a higher risk for contamination given the long protocols and manipulations associated with them (PDMS preparation and Glass coverslip cleaning).

### Potential solution

To reduce the risk of contamination, PDMS substrates should be handled exclusively under sterile conditions, such as under a laminar flow hood or in a clean room. Further, after PDMS preparation (PDMS preparation, step 20) the PDMS may be submerged for 30 min in 70 % ethanol, rinsed 3 times with sterile water, dried for 1 h, exposed to germicidal ultraviolet light, and finally autoclaved together with the pre-cleaned glass coverslip. The autoclaving (Glass coverslip cleaning, step 26) represents the final sterilization of PDMS and glass coverslip before plating the cells. Additionally, we note that sonication is a strategy that can be implemented to improve sterility and long-term biocompatibility of PDMS with neurons by eliminating possible monomers or post unmolding residues. In our experiments, no differences in neuronal network health and overall behavior were observed when sonication was applied as compared to our standard (sonication-free) preparation. Sonication is a truly fundamental step if PDMS substrates need to be reused, with non-sonicated reused PDMS substrates leading to very poor adhesive protein coating and overall deficient culture development.

### Problem 2: PDMS detachment from the glass coverslip

Premature detachment of the PDMS casts from the glass coverslip can compromise the quality of the neuronal culture or make it unusable (Glass coverslip cleaning).

### Potential solution

To ensure proper adhesion, both surfaces must be completely dried before placing PDMS on the glass. For a stronger bond, oxygen plasma can be applied to the surfaces of PDMS for 30 s at high power and 0.8 Torr, followed by immediate attachment of the PDMS onto the glass coverslip as in step 25 (Glass coverslip cleaning).

### Problem 3: Excessive aggregation

**Figure 5:**
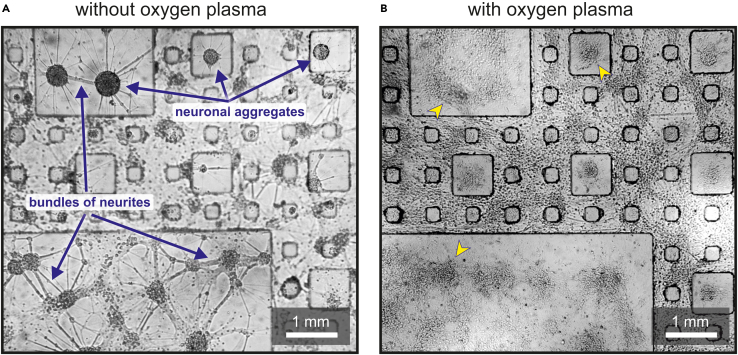
Aggregation in PDMS topographical patterns (A) Bright-field image of rat primary neuronal cultures at DIV 7 without oxygen plasma treatment and therefore a hydrophobic surface, leading to the formation of highly dense aggregates of neurons (dark spherical objects) connected through bundles of neurites (thick straight filaments). The formed network is highly arbitrary both in the position and interconnectivity of the aggregates and is largely insensitive to the PDMS topography. (B) Oxygen plasma procured a hydrophilic surface that favored adhesive protein coating, leading to a neuronal network in which single cells are visible and cover the surface of the PDMS more homogeneously, although some aggregation persists (yellow arrowheads).

Neurons form aggregates of densely packed neurons during the first days after plating, masking the capacity of PDMS motifs to guide connectivity or to restrict the positions of the neurons (Figure 5A). These aggregates are formed when the pulling forces between neurons overcome their adhesion to the substrate. The presence of aggregates is therefore associated with failed coating of adhesive proteins or a progressive coating depletion over time. Aggregates emerge as a self-organized process and, in extreme conditions, they appear under the microscope as spherical objects interconnected through bundles of neurites,^18^^,^^19^ with the number of aggregates and their interconnectivity difficult to control. We observed that dense aggregates essentially ignore the PDMS topographical modulation, possibly because their diameter of about 100–200 microns is on the order of the topographical motifs (typically 50–100 microns deep). This is illustrated in Figure 5A, which highlights the random distribution of cell aggregates and their arbitrary interconnectivity (Culturing cells atop the PDMS patterned substrates).

### Potential solution

Apply oxygen plasma treatment to enhance coating of adhesive proteins and maximize the uniform distribution of single cells over the surface of the PDMS motifs. Make sure that the time spanned from oxygen plasma treatment to adhesive protein coating is not excessive, and that the protein concentration is sufficiently high. In this regard, coating may be supplemented with mouse laminin to strengthen adhesion (Figure 5B). For that, in step 30, wash the PDL-coated PDMS three times with sterile Milli-Q water, then add laminin, and incubate the PDMS substrate overnight at 37°C. Next day, remove residual laminin from the well and add the cells to be cultured. It is also recommended to verify under the microscope that neuronal dissociation is correct, i.e., that there are no clumps of neurons, and to explore different cell plating densities to obtain the optimal one that maximizes a homogeneous distribution of neurons. Both an excess and a deficit of neurons may lead to cell aggregation. We remark that, in general, neuronal cultures free of aggregation are very difficult to produce, as illustrated in Figure 5B by the clear presence of aggregated areas (yellow arrowheads).

### Problem 4: Areas in the culture without neurons

Some regions of the neuronal network appear empty of neurons (related to step 29 in culturing cells atop the PDMS patterned substrates).

### Potential solution

This problem indicates that neurons failed to attach and develop in those regions, and it is typically related to an inhomogeneous coating of adhesive proteins. A solution is to apply a gentle vacuum to the wells where neurons will be cultured just after introducing the solution containing the adhesive proteins. Vacuum will eliminate the air bubbles trapped in the smallest motifs of the PDMS patterns.

### Problem 5: Calcium imaging

Inadequate signal detection during Ca^2+^ imaging experiments, typically characterized by low fluorescence intensity or a poor signal-to-noise ratio, can substantially hinder the reliable monitoring of neuronal activity and the characterization of network dynamics. In addition, in some cases, excessively thick PDMS layers may also interfere with effective light transmission (Recording neuronal activity).

### Potential solution

First, PDMS topographical casts should be as thin as possible to maximize light transmission. However, very thin PDMS (below 500 μm thick) is fragile and difficult to unmold and handle, so a compromise should be made. Second, neuronal activity recordings on glass or flat PDMS should be conducted before using topographical PDMS to ascertain the optimal fluorescence imaging parameters, which often require to balance the area of observation, camera settings and sensitivity, the temporal resolution of acquired data, and the health of the neurons. For instance, signal-to-noise ratio of the fluorescence signal can be enhanced by increasing the intensity of excitation light, the camera exposure time, and by mounting microscope objectives specialized for fluorescence. However, strong fluorescence light may compromise neurons health, and high exposure times may hinder the detection of processes at relevant time scales. The selection of the adequate GECI and serotype is also very important. Extensive literature search for available GECIs, preliminary tests and accessibility to vector facilities are important factors that should be taken into account to ensure optimal results.

## Resource availability

### Lead contact

Further information and requests for resources should be directed to the lead contact, Jordi Soriano (jordi.soriano@ub.edu).

### Technical contact

Technical questions on executing this protocol should be directed to and will be answered by the technical contact, Mireia Olives-Verger (mireia.olives@ub.edu).

### Materials availability

This study did not generate new unique reagents but produced computer designs of the black-and-white motifs that were transferred to the topographical resin molds and PDMS casts. These computer designs can be provided upon request.

### Data and code availability

This study did not generate new data and unique codes.

## Acknowledgments

This research was supported by the European Union Horizon 2020 research and innovation program under grant no. 964877 (project NEU-CHiP). J.S. and M.O.-V. acknowledge support from grant PID2022-137713NB-C22, funded by MCIU/AEI/10.13039/501100011033, ERDF/EU, and the Generalitat de Catalunya under grant 2021-SGR-00450. The authors thank the Scientific and Technological Centers (CCiTUB), Universitat de Barcelona, and staff for their support and advice on resin mold fabrication, soft lithography, and confocal microscopy. Specifically, the authors would like to thank Albert Rigat Pujolàs, Teresa Galán Cascales, Sandra Segura Feliu, and Emma Oriol Ferrer.

## Author contributions

J.S. conceived the experiments and framework. J.S. and M.O.-V. designed the patterns. M.O.-V. fabricated the master molds. A.-C.H. and M.O.-V. carried out the PDMS manipulation and initial cell culturing. A.-C.H. performed the immunofluorescent labeling. M.O.-V., A.-C.H., and J.S. wrote the initial draft. J.S. edited and reviewed the manuscript.

## Declaration of interests

The authors declare no competing interests.
